# Mass Spectrometric Analysis of *N*-Glycome of Patatin Proteins from Three Potato Cultivars

**DOI:** 10.3390/molecules31152591

**Published:** 2026-07-24

**Authors:** Lingmei Li, Sidi Luo, You Wu, Yajuan Zhou, Yongjie Ma, Jiangxiu Niu

**Affiliations:** Luoyang Key Laboratory of Organic Functional Molecules, College of Food and Drug, Luoyang Normal University, Luoyang 471934, China; 19179748121@163.com (S.L.); 15121680623@163.com (Y.W.);

**Keywords:** potato, cultivar, patatin, *N*-glycome, LC-UV-MS/MS

## Abstract

As a vital post-translational modification of potato tuber proteins, *N*-glycosylation directly regulates protein structural stability, biological activity and processing properties. *N*-glycome profiles of tuber storage proteins exhibit cultivar-specific characteristics, but the differential glycosylation across potato germplasms has not been fully elucidated. In this work, the major tuber patatin glycoprotein was purified from three potato cultivars as research material to investigate their *N*-glycosylation features and inter-cultivar variations. *N*-glycans were released via nonreductive chemical cleavage and derivatized with PMP, followed by qualitative and quantitative profiling using liquid chromatography-tandem mass spectrometry (LC-UV-MS/MS). In total, 11, 6 and 3 distinct *N*-glycan structures were identified from the three tested cultivars. Two novel glycan structures, Man_3_XylGlcNAc_2_ and Man_4_XylGlcNAc_2_, were identified in this study. The relative abundances of Man_3_XylFucGlcNAc_2_ and Man_3_XylGlcNAc_2_ exhibited highly significant inter-cultivar differences (*p* < 0.01). Notably, patatin carried unique *N*-glycans modified with exclusive α1,3-fucosylation, and overall glycan composition and relative abundance varied markedly across the three cultivars. This study systematically elucidates the effects of potato cultivar germplasm on patatin *N*-glycosylation and analyzes the unique glycosylation signatures of patatin from the three varieties. The obtained glycomic dataset provides fundamental theoretical support for germplasm screening, functional modification of potato storage proteins, and the optimization of potato deep-processing.

## 1. Introduction

Protein glycosylation is a ubiquitous post-translational modification in eukaryotes, in which *N*-glycans are sequentially processed through the endoplasmic reticulum–Golgi pathway through cleavage, modification, and elongation, forming structures such as high-mannose, hybrid, and complex types [1,2,3]. This process directly participates in protein folding, intracellular transport, molecular recognition, and signal regulation, serving as a core component that determines glycoprotein function and biological activity. Plant *N*-glycans possess conserved and unique structural features, commonly containing core β-1,2-xylose and α-1,3-fucose modifications, with some species bearing the Lewis A epitope [4,5]. These characteristics are closely related to plant growth and development, stress responses, as well as food allergies, immune cross-reactions, and the safety of therapeutic proteins expressed in plant systems [6,7,8].

Potato (*Solanum tuberosum* L.) is the fourth most prominent staple crop worldwide, alongside its widespread use as a dietary vegetable. Its tubers are rich in starch, protein, vitamins and minerals, and the crop plays a vital role in food security, food processing and dietary nutritional demands [9,10]. Potato possesses abundant germplasm resources, and distinct differences in tuber morphology, taste, nutritional composition, stress tolerance and processing quality are widely observed among various cultivars, landraces and breeding materials [11]. Such phenotypic diversity is jointly determined by differences in genetic background, metabolic regulation and post-translational modifications [7,12,13]. The Solanaceae family is a group of crops with extremely high economic value, and thus extensive *N*-glycome investigations have been conducted on tomato and pepper. In ripening tomato fruit, the predominant glycan Man_3_FucXylGlcNAc_2_ accumulates progressively while Man_6_–Man_9_ high-mannose glycans decline, consistent with rising Golgi glycosyltransferase activity [14]. Suppression of Golgi class I α-mannosidase causes massive high-mannose glycan buildup, impairing fruit maturation and seed vitality. For non-climacteric pepper fruit, α-mannosidase governs *N*-glycan maturation and fruit softening; natural germplasm-specific disparities in mannosidase activity drive differential high-mannose glycan enrichment, which further correlates with glycoprotein trafficking efficiency and postharvest shelf life [15].

Patatin glycoprotein is the major storage protein and primary allergen carrier in potato tubers, and its *N*-glycan composition directly determines protein stability, allergenic potential and functional characteristics [16,17,18,19]. Previous studies have demonstrated obvious interspecific divergence in the *N*-glycome among Solanaceous crops [2,20]. In potatoes, genotypic variations across different cultivars alter the expression levels of glycosyltransferases and the efficiency of glycan maturation [11,21], thereby resulting in cultivar-specific disparities in *N*-glycan composition, relative abundance and structural isomer distribution [16]. Nevertheless, most current investigations on potato *N*-glycans are confined to individual cultivars. Systematic comparative analyses of *N*-glycan profiles among diverse potato varieties, the underlying mechanisms driving their structural diversity, and their regulatory association with key quality traits remain poorly understood [7,12].

With the rapid development of glycomics technologies, high-throughput profiling of plant *N*-glycan profiles has become feasible, providing technical support for revealing glycosylation differences among varieties [22,23,24,25]. HILIC-LC-MS-based comparative glycomics has become the standard workflow to profile genotype-, tissue- or stress-induced glycosylation shifts [26]. Comparative studies on *N*-glycans of different potato varieties can clarify the varietal diversity and structural characteristics of the potato *N*-glycome [27,28,29]. It also provides references for screening low-allergenic potato varieties [30].

Accordingly, three potato cultivars were selected as experimental materials in this work to systematically characterize and compare the compositional profiles, relative abundance, and structural variations in tuber *N*-glycans. We further explored inter-cultivar glycan diversity, with the goal of offering theoretical fundamentals and empirical data to support quality potato breeding, food allergen safety assessment, and basic glycoscience research.

## 2. Results and Discussion

### 2.1. Isolation, Purification and Identification of Patatin

**Figure 1:**
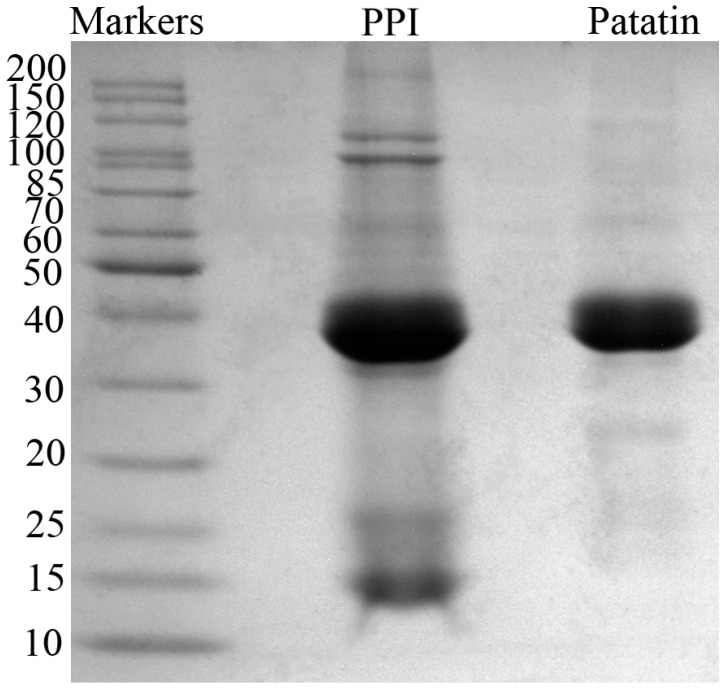
Profiling of eluted proteins by SDS-PAGE.

The study presented in this paper purified patatin using DEAE-52 and ConA Sepharose 4B affinity chromatography. The purity and molecular weight of the isolated patatin were evaluated by 12% SDS-PAGE. As shown in Figure 1, the molecular weight markers ranged from 10 to 200 kDa. Patatin is a major storage protein in potato tubers. The potato protein isolate (PPI) fraction displayed a prominent band at ~40 kDa, which is characteristic of patatin, alongside additional faint bands at lower (~15 kDa) and higher (~100 kDa) molecular weights, corresponding to other endogenous potato proteins. The purified patatin preparation showed a single, sharp band at ~40 kDa, confirming the successful removal of contaminating proteins and the high homogeneity of the purified patatin, which is consistent with the typical molecular weight of monomeric patatin reported in previous studies [31,32,33,34,35]. No detectable degradation products or aggregates were observed in the patatin lane, demonstrating the effectiveness of the purification protocol. In comparison with traditional purification methods that usually produce multiple bands caused by protein glycosylation heterogeneity and partial degradation, the optimized protocol in this study significantly improved the purity and structural integrity of the obtained patatin.

### 2.2. ESI-MS and MS/MS Analysis of Patatin Glycans

**Figure 2:**
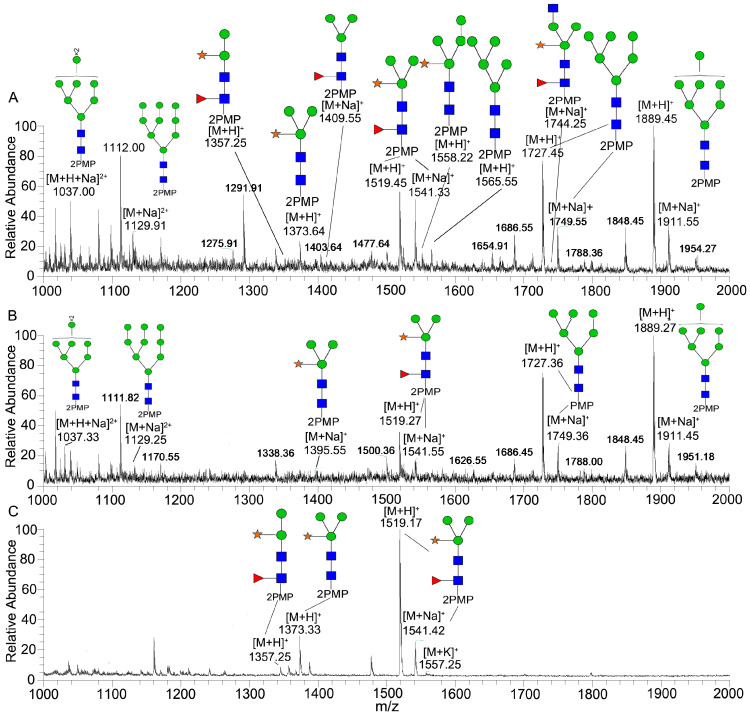
ESI-MS profiles of the *N*-glycans released from patatin. (**A**) is the Gan Nong Shu No. 7 (Wuwei, China) variety, (**B**) is the Ke Xin No. 1 (Qiqihar, China) variety, and (**C**) is the Favorita (Qingdao, China) variety. Symbol nomenclature: 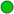, mannose; 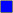, *N*-acetylglucosamine; 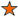, xylose; 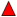, fucose.

PNGase F and PNGase A have been widely used for the cleavage of *N*-glycans from glycoproteins, but PNGase F could not release core α1,3-fucosylated *N*-glycans [36] and PNGase A exhibited low deglycosylation efficiency [37,38]. Therefore, PNGase A was not adopted in our formal experiments. For the discrimination of plant *N*-glycans, only PNGase F digestion and chemical deglycosylation were used as two parallel glycan release approaches in this study. Our laboratory developed a new chemical method for nonreductive *N*-glycan release and simultaneous labeling with PMP in one pot that has no selectivity to different types of *N*-glycans [39]. The new PMP derivatization method directly labels *N*-glycans after alkali hydrolysis of glycosidic bonds with NaOH (one-pot method). Potential side reactions, derivatization efficiency, and glycan recovery rates were strictly controlled in this study. All samples were processed with identical derivatization conditions and unified purification procedures to minimize technical variations, ensuring high reliability and reproducibility of the relative quantification results. In this paper, a qualitative analysis of 2-PMP-derivatized *N*-glycans from patatin of three different potato varieties was performed with ESI-MS^n^ [39]. The results show that the *N*-glycans of all three varieties are predominantly complex-type (Figure 2). Additionally, plant-specific complex glycans modified with fucose and xylose were detected. However, there are significant differences among the three varieties in terms of glycan types, signal intensity, and dominant glycan types.

Figure 2A include five high-mannose-type glycans (Man_5_GlcNAc_2_(PMP)_2_, Man_6_GlcNAc_2_(PMP)_2_, Man_7_GlcNAc_2_(PMP)_2_, Man_8_GlcNAc_2_(PMP)_2_ and Man_9_GlcNAc_2_(PMP)_2_) and six complex glycans (Man_2_FucXylGlcNAc_2_(PMP)_2_, Man_3_XylGlcNAc_2_(PMP)_2_, Man_3_XylFucGlcNAc_3_(PMP)_2_, Man_3_FucGlcNAc_2_(PMP)_2_, Man_3_FucXylGlcNAc_2_(PMP)_2_, Man_4_XylGlcNAc_2_(PMP)_2_); Figure 2B includes four high-mannose-type glycans (Man_6_GlcNAc_2_(PMP)_2_, Man_7_GlcNAc_2_(PMP)_2_, Man_8_GlcNAc_2_(PMP)_2_ and Man_9_GlcNAc_2_(PMP)_2_) and two complex glycans (Man_3_XylGlcNAc_2_(PMP)_2_, Man_3_FucXylGlcNAc_2_(PMP)_2_); and Figure 2C includes three complex glycans (Man_2_FucXylGlcNAc_2_(PMP)_2_, Man_3_XylGlcNAc_2_(PMP)_2_, Man_3_FucXylGlcNAc_2_(PMP)_2_). Isomers may exist for *m*/*z* 1357, 1409 and 1541. Most of the molecular ion peaks were assigned to [M + Na]^+^, although a few peaks were assigned to [M + H]^+^, [M + K]^+^, ([M + H + Na]^2+^) and [M + 2Na]^2+^. Among them, the ion at m/z 1037 represents Man_8_GlcNAc_2_(PMP)_2_ ([M + H + Na]^2+^) and 1129 represents Man_9_GlcNAc_2_(PMP)_2_ ([M + 2Na]^2+^); the ions at *m*/*z* 1357, 1373, 1519, 1558, 1565, 1727 and 1889 ([M + H]^+^) represent Man_2_FucXylGlcNAc_2_(PMP)_2_, Man_3_XylGlcNAc_2_(PMP)_2_, Man_3_FucXylGlcNAc_2_(PMP)_2_, Man_4_XylGlcNAc_2_(PMP)_2_, Man_5_GlcNAc_2_(PMP)_2_, Man_6_GlcNAc_2_(PMP)_2_ and Man_7_GlcNAc_2_(PMP)_2_; the ions at *m*/*z* 1395, 1409, 1541, 1744, 1749 and 1911 ([M + Na]^+^) represent Man_3_XylGlcNAc_2_(PMP)_2_, Man_3_FucGlcNAc_2_(PMP)_2_, Man_3_FucXylGlcNAc_2_(PMP)_2_, Man_3_FucXylGlcNAc_3_(PMP)_2_, Man_6_GlcNAc_2_(PMP)_2_, Man_7_GlcNAc_2_(PMP)_2_; and the ion at *m*/*z* 1557 ([M + K]^+^) represents Man_3_FucXylGlcNAc_2_(PMP)_2_. It is already known that PNGase F is ineffective against core α1,3-fucosylated *N*-glycans, while the PMP derivation method has no selectivity for either isomer [36,39]. Therefore, the two methods were employed here to differentiate α1,3- and core α1,6-fucosylated *N*-glycans. No core α1,6-fucosylated *N*-glycans were detected among the *N*-glycans released by PNGase F from the patatin protein (Figure S1B). However, core α1,3-fucosylated *N*-glycans were observed in patatin protein *N*-glycans released by the PMP derivation method (Figure S1A). All PMP-labeled glycans were identified by MS/MS (Figure S2). Through MS/MS analysis, it was found that all three samples contained α1,3-fucosylated *N*-glycans.

In previous studies, patatin was confirmed to possess only *N*-glycans with α1,3-fucosylation [16,19,40]. Previous studies have shown that *N*-glycans differ among potato varieties. The *Solanum stenotomum* Juz. & Bukasov variety contains eight *N*-glycans, and the glycans Man_2_XylGlcNAc_2_ and Man_3_FucXylGlcNAc_4_ were not found in our study, while other varieties contain few glycans [16]. In contrast, a total of 11 *N*-glycans were detected in our analysis in Gan Nong Shu No. 7 (Gansu, China) varieties, among which five structures had not been previously reported for patatin in reference Lattová E [16], including Man_3_XylGlcNAc_2_(PMP)_2_, Man_3_XylGlcNAc_2_(PMP)_2_, Man_5_GlcNAc_2_(PMP)_2_, Man_6_GlcNAc_2_(PMP)_2_, and Man_9_GlcNAc_2_(PMP)_2_. The innovative findings of this work focus specifically on the complex-type and xylose-modified (Man_3_XylGlcNAc_2_(PMP)_2_, Man_3_XylGlcNAc_2_(PMP)_2_) *N*-glycan structures of patatin, which have rarely been characterized in prior studies [16]. The Ke Xin No. 1 (Heilongjiang, China) variety has six *N*-glycans, and the Favorita (Shandong, China) variety has three *N*-glycans. These results reveal higher glycosylation heterogeneity in patatin than previously acknowledged. The differences in glycan profiles may result from variations in potato varieties, cultivation environments, or the superior resolution and sensitivity of the analytical approaches adopted in this study. The newly identified fucosylation patterns and *N*-glycan structures also provide new clues for further exploring the structure–function relationship of patatin.

### 2.3. Separation and Quantitative Analysis of Patatin N-Glycans by Online HILIC-ESI-MS/MS

**Figure 3:**
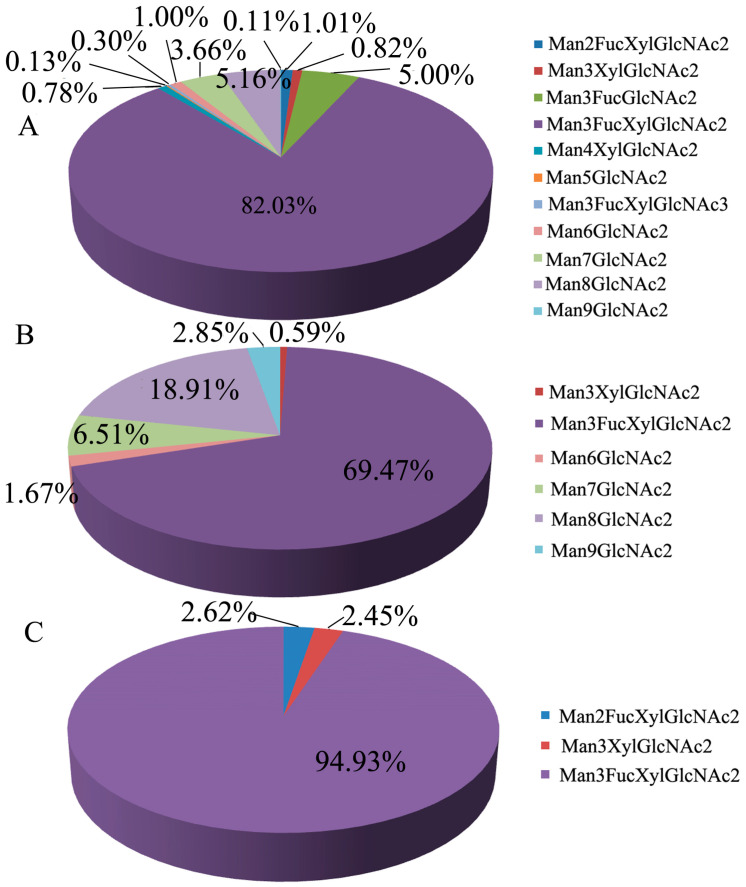
Chart showing the quantitative distribution of the *N*-glycans of patatin. (**A**) is the Gan Nong Shu No. 7 (Wuwei, China) variety, (**B**) is the Ke Xin No. 1 (Qiqihar, China) variety, and (**C**) is the Favorita (Qingdao, China) variety. Notes: Man, mannose; GlcNAc, *N*-acetylglucosamine; Xyl, xylose; Fuc, fucose.

Isomers exist extensively within the glycans released from glycoproteins. Online HILIC-UV-MS/MS^n^ analysis of the *N*-glycans derived from patatin glycoproteins was performed to separate possible *N*-glycan isomers and obtain information about their distributions and abundance. Three replicate experiments were conducted for each sample variety. The obtained extracted ion chromatograms (EICs) of these *N*-glycans are shown in Figure S3. Their qualitative information (the mass accuracy (ppm)) is summarized in Table 1 and Table S1, and quantitative information is presented in Figure 3 and Figure S4.

As presented in Figure S3, the online HILIC-ESI-MS EICs provide good profiling of the *N*-glycoforms of patatin glycoprotein, especially the possibly existing isomers of these *N*-glycans. Briefly, a total of 11 peaks were identified in Gan Nong Shu No. 7 (Gansu, China). For Ke Xin No. 1 (Heilongjiang, China), six peaks were observed. Favorita (Shandong, China) yielded three peaks. The 11 identified *N*-glycans exhibited characteristic peaks at *m*/*z* 1395.3, 1357.3, 1409.3, 1541.3, 1587.3, 1558.2, 1744.3, 1749.3, 1911.3, 1037.3 and 1129.3, with corresponding retention times of 35.87, 36.41, 45.61, 53.50, 51.74, 56.81, 62.05, 63.08, 73.35, 82.58 and 89.16 min, respectively. These observations matched the structural features of *N*-glycans released by PNGase F (Figure S1). Most importantly, five new *N*-glycans (two xylose-modified) were identified on patatin for the first time in this study.

To address overlapping signals from co-eluting isomeric glycan species, we combined UV peak profiling and mass spectral matching to separate and annotate individual isomers. Specifically, each unresolved UV peak was subdivided into distinct isomeric components according to its unique accurate *m*/*z* signature. Retention time calibration standards were used to precisely align UV chromatographic traces and LC-MS raw data, establishing one-to-one correspondence between UV quantitative peaks and MS structural identifications. The relative abundance of each glycan was calculated from integrated UV peak areas under the assumption of uniform molar UV response among all PMP-labeled glycans. To quantify the distribution of different *N*-glycan types on patatin from three potato cultivars, their relative abundances were calculated based on the integrated UV peak areas from HPLC analysis, and the results are summarized in Figure 3.

For Gan Nong Shu No. 7 (Gansu, China), the patatin contained high-mannose (Man_5_GlcNAc_2_, Man_6_GlcNAc_2_, Man_7_GlcNAc_2_, Man_8_GlcNAc_2_ and Man_9_GlcNAc_2_; 10.03%), complex (Man_2_FucXylGlcNAc_2_, Man_3_XylGlcNAc_2_, Man_3_XylFucGlcNAc_3_, Man_3_FucGlcNAc_2_, Man_3_FucXylGlcNAc_2_, Man_4_XylGlcNAc_2_; 89.94%) *N*-glycans (Figure 3A). Patatin from Ke Xin No. 1 (Heilongjiang, China) consisted of 29.95% high-mannose (Man_6_GlcNAc_2_, Man_7_GlcNAc_2_, Man_8_GlcNAc_2_ and Man_9_GlcNAc_2_) and 70.05% complex (Man_3_XylGlcNAc_2_, Man_3_FucXylGlcNAc_2_) *N*-glycans (Figure 3B). Favorita (Shandong, China) only produced complex-type *N*-glycans (Man_2_FucXylGlcNAc_2_, Man_3_XylGlcNAc_2_, Man_3_FucXylGlcNAc_2_), which accounted for 100% of total glycans (Figure 3C). In order to more clearly elucidate the difference among the occupancy rates of each glycan in different patatin protein samples, their occupancy rate data are also comparatively summarized in Figure S4. Man_3_FucXylGlcNAc_2_ is a common dominant glycan shared by the three groups (Figure S4). However, the relative content of this core β1,2-xylose and α1,3-fucose complex *N*-glycan shows extremely significant differences among the three groups (*p* < 0.01). The abundance follows the order C group > A group > B group. In particular, this glycan accounts for nearly 95% in the C group, which is much higher than in the A and B groups. For low-abundance branched glycoforms, Man_2_FucXylGlcNAc_2_ shows significant differences between A and B (*p* < 0.01); Man_3_XylGlcNAc_2_ shows extremely significant differences among the three groups (*p* < 0.01); and Man_7_GlcNAc_2_, Man_8_GlcNAc_2_, and Man_9_GlcNAc_2_ show significant differences between A and B (*p* < 0.01). This indicates that there are biological differences in the *N*-glycosylation modification types among the three groups of samples. Man_3_FucXylGlcNAc_2_, the high-mannose chains of the series, and Man_3_FucGlcNAc_2_ can serve as characteristic glycan biomarkers to distinguish groups A, B, and C. This also provides a glycomics basis for a subsequent analysis of the differential expression regulation of glycosyltransferases and mannosidases, as well as the functional association mechanisms of glycoproteins in different samples.

Overall, α1,3-fucosylated glycans excluded the *N*-glycan profiles of patatin in all three cultivars. Comparative analysis indicated that Favorita had the highest relative content of complex-type *N*-glycans. The distinct glycan compositions revealed evident cultivar-specific variations in patatin *N*-glycosylation [16,41]. Such discrepancies in glycan distribution are largely attributed to the different genetic backgrounds and natural growth conditions of potato varieties [16]. As plant-derived complex-type *N*-glycans are typically characterized by β1,2-xylose and α1,3-fucose residues [16,42], the exclusive presence of complex-type glycans in Favorita suggests a unique glycosylation regulatory mechanism. These findings deepen our understanding of glycosylation heterogeneity among patatins derived from different potato germplasms.

Herein, we purified patatin from three potato cultivars and systematically analyzed its *N*-glycosylation characteristics. Eleven *N*-glycans were identified, five of which were unreported structures. Quantitative results showed great variations in the distribution of high-mannose, complex and hybrid *N*-glycans as well as fucosylation levels across different cultivars. Favorita possessed 100% complex-type *N*-glycans, with α1,3-fucosylation as the major modification in all samples. These results verify that the *N*-glycosylation of patatin is closely related to potato genotypes and cultivation conditions. This study supplements the glycosylation data of patatin and enriches the understanding of its glycosylation heterogeneity in potato germplasms.

## 3. Materials and Methods

### 3.1. Reagents and Instruments

Potato (*Solanum tuberosum* L.) was purchased from Luoyang Farmers’ Market (Henan Province, Luoyang, China); Gan Nong Shu No. 7 (Wuwei, China), Ke Xin No. 1 (Qiqihar, China) and Favorita (Qingdao, China) were the three varieties. MW markers of proteins were obtained from Fermentas (Burlington, ON, Canada). Sodium dodecyl sulfate (SDS), 1-phenyl-3-methyl-5-pyrazolinone (PMP), and DL-dithiothreitol (DTT) were purchased from Shanghai aladdin company (Shanghai, China), and other reagents were analytically pure. The MD34 (retention MW: 8000–14,000) dialysis membrane was from Union Carbide Co. (Danbury, CT, USA). Concanavalin A Sepharose 4B and DEAE-52 packing were purchased from General Electric Healthcare (Fairfield, CT, USA). The C18 solid phase extraction column was purchased from Sigma Aldrich Co. (St. Louis, MO, USA). The experimental water was prepared in a Milli-Q system (Millipore, Burlington, MA, USA). Electrospray ionization mass spectrometry (ESI-MS) and high-performance liquid chromatography (HPLC) were performed using instruments produced by Thermo Scientific (Thermo Scientific, Waltham, MA, USA). A TSK-GEL amide-80 column (4.6 mm × 250 mm, 5 μm) was purchased from Tosoh Company (Tokyo, Japan).

### 3.2. Preparation of Potato Protein Isolate

Potato protein isolate (PPI) was prepared by isoelectric point acid precipitation [43]. Firstly, fresh potatoes were cleaned and sliced. Subsequently, 4 L of distilled water was added per kilogram of fresh potatoes, followed by sodium bisulfite supplementation to reach a final concentration of 0.12%. After refining, the raw material was stirred and extracted at room temperature for 2 h. The mixture was filtered using double-layer gauze, and the resulting filtrate was centrifuged at 3000× *g* and 4 °C for 15 min. The collected supernatant was acidified to pH 4.0 with 1 mol/L hydrochloric acid, followed by 10 min of magnetic stirring and static settling for 1 h. The sediment was redissolved in distilled water and neutralized to pH 7.0. After 24 h of dialysis, the solution was freeze-dried to yield potato protein isolate (PPI), which was preserved at −20 °C for subsequent experiments.

### 3.3. Purification of Patatins

A total of 1 g PPI was extracted using 25 mM Tris-HCl (pH 7.4) buffer at a solid-to-liquid ratio of 1:10 (g/mL). The extraction was carried out at 4 °C for 30 min, followed by centrifugation at 4 °C and 8000 g for 15 min to collect the supernatant. Ion-Exchange chromatography was completed on DEAE 52-Cellulose (15 mL, GE Healthcare, Chicago, IL, USA) with a starting 25 mM Tris-HCl buffer (90 mL, pH 7.4). After applying the protein supernatant (10 mL), the column was washed with 60 mL of the starting buffer to remove unbound proteins, and isocratic elution of bound proteins was performed with 30 mL of elution buffer (25 mM Tris-HCl pH 7.4 and 0.5 M NaCl), after which the wash solution was collected. Affinity chromatography was performed on Concanavalin A Sepharose 4B (10 mL, GE Healthcare, USA). The column was equilibrated with 60 mL of starting buffer (25 mM Tris-HCl, pH 7.4, and 0.5 M NaCl). The wash solution from the DEAE-52 cellulose was then adsorbed onto the Concanavalin A Sepharose 4B affinity chromatograph [34]. After, the column was washed with 100 mL of buffer (a mixture of 25 mM Tris-HCl, pH 7.4, and 0.5 M NaCl) to remove unbound proteins. Elution of bound patatins was then performed with 30 mL elution buffer containing 25 mM Tris-HCl, pH 7.4, 0.5 M NaCl, and 100 mM α-methyl-D-glucoside. The eluted patatins were collected, dialyzed using the MD34 (retention MW: 8000–14,000) dialysis membrane to remove salts, and freeze-dried and stored at −20 °C.

### 3.4. Purity Identification by SDS-PAGE

Sodium dodecyl sulfate-polyacrylamide gel (SDS-PAGE) was performed by Laemmli [44], using 5% acrylamide in stacking gels and 12% acrylamide in separating gels. A total of 2 mg of PPI and patatin protein sample was dissolved in 500 µL of loading buffer (1% SDS, 40% glycerol, 0.1% dithiothreitol, and 0.05% bromophenol blue in 10 mM Tris-HCl buffer, pH 8.0), then heated at 100 °C for 10 min and cooled afterwards. For maximum visualization of PPI and patatin, the amount of protein loaded was 10 μg for all samples. SDS-PAGE molecular weight standards ranging from 15 to 200 kDa were used. Electrophoresis was carried out at 90 V in the stacking gel and 120 V in the separating gel using a Tris-Glycine electrophoretic buffer containing 0.1% SDS (pH 8.3). After electrophoresis, the gel was stained with 0.1% Coomassie Brilliant Blue G-250 staining solution (45% methanol, 45% water, and 10% acetic acid) for 30 min. Subsequently, the gel was destained with a solution containing 1.31 mol/L acetic acid and 1.23 mol/L methanol over 12 h. Gel images were captured using a Bio-Rad GelDoc 2000 imaging system (Hercules, CA, USA), and band analysis was performed with Gel-Pro Analyzer 4.0 software.

### 3.5. Release of N-Glycans from Patatin by PNGase F

Patatin protein (2–5 mg) was dissolved in protein denaturing solution (500 μL) containing 5% SDS and 0.4 M DTT and incubated at 100 °C for 10 min. When the sample was cooled, 50 μL of sodium phosphate buffer (0.5 mol/L, pH 7.5), 50 μL of 10% aqueous NP-40, and 1 μL of PNGase F (500 units) solution were sequentially added, followed by incubation at 37 °C for 24 h. Subsequently, the sample was boiled for 10 min to terminate the enzymatic reaction and then loaded onto a Sep-Pak C18 SPE column. Elution of *N*-glycans was performed with 15 mL of water. Desalting of *N*-glycans was achieved using a Carbograph SPE column (Deerfield, IL, USA). The column was washed with 3 mL of water to remove salts, and the *N*-glycans were eluted with 25% aqueous acetonitrile solution. The eluates were concentrated to dryness under reduced pressure for further use.

### 3.6. Nonreductive Chemical Release and Simultaneous Labeling of Glycoprotein N-Glycans

The operation was performed according to a method developed in our laboratory [40]. Three independent biological replicate experiments were carried out on patatins from each cultivar. Briefly, 2–5 mg of patatin protein was suspended in 4 mL of 0.5 M aqueous NaOH solution containing 0.7 M PMP prior to the addition of 2 mL of methanol to the sample. The obtained mixture was incubated at 75 °C for 32 h. When the reactions were completed, the sample was neutralized with glacial acetic acid and washed three times with 4 mL of dichloromethane to remove excess PMP. The obtained sample solution was concentrated to dryness to remove methanol and excess acetic acid. After dissolving the sample in 1 mL of water, it was loaded onto a Sep-Pak C18 column, washed with 6 mL of water to remove salts, and 3 mL of *N*-glycans were eluted with 25% acetonitrile. The eluates were concentrated to dryness for further analysis.

### 3.7. Analysis by ESI-MS and MS/MS

The ESI-MS analysis of the glycans was performed on an LTQ XL linear ion trap mass spectrometer coupled with an electrospray ion source and an HPLC system (Thermo Scientific, USA). Each glycan sample was dissolved in 500 μL of water or 50% aqueous methanol (vol/vol) and directly infused into the mass spectrometer via a 2-μL Rheodyne loop. The infused glycans were brought into the electrospray ion source using a stream of 50% methanol at a flow rate of 20 μL/min. The voltage was 4 KV, the sheath gas (N_2_) flow rate was 20.0 arb, the auxiliary gas (N_2_) flow rate was 10.0 arb, the capillary lens voltage was 250 V, and the capillary voltage and temperature were 350 V and 300 °C. For analysis by tandem mass spectrometry (MS/MS), the glycans were subjected to fragmentation by collision-induced dissociation (CID), with helium (He) as the collision gas. Collision parameters were left at default values with a normalized collision energy degree of 30 and an isotope width of *m*/*z* 3.00. The activation Q was set at 0.25 and the activation time at 30 ms. The MS and MS/MS data were recorded using the LTQ Tune (version 2.7.0) software. The structure of each glycan was assigned according to MS/MS data combined with computational analysis using the GlycoWorkbench (version 1.1) software and knowledge of *N*-glycan biosynthesis [45].

### 3.8. Online HILIC-UV-MS/MS Analysis

Online HILIC-UV-MS/MS analysis of PMP-labeled glycans was also performed on the HPLC-ESI-MS system (Thermo Scientific, USA) using a TSK-GEL amide-80 column (4.6 mm × 250 mm, 5 μm) (Tosoh Corporation, Tokyo, Japan). Each glycan sample was dissolved in 20 μL of deionized water, and 10 μL of the sample solution was injected by an autosampler. The elution gradient was as follows: solvent A, acetonitrile; solvent B, 10 mmol/L aqueous ammonium acetate (pH 6.0); time = 0 min (t = 0 min), 80% A, 20% B, 1 mL/min; t = 120 min, 60% A, 40% B, 1 mL/min. The fractions eluted from the chromatographic column were directly imported into the ESI-MS system for detection. For the ESI-MS system, a rapid alternation mode between the segments of positive ion mode MS and MS-dependent MS/MS was adopted. For the MS-dependent MS/MS, the normalized collision energy was set at 30, and the lowest signal intensity was set at 500. The other parameters used for MS and MS-dependent MS/MS were the same as those described above. Data acquisition was performed using Xcalibur (version 2.2.42) software (Thermo, Waltham, MA, USA). The obtained data were manually interpreted, and the proposed glycan compositions and sequences were checked using GlycoWorkbench (version 1.1) software. The area values of the chromatographic peaks of glycans were used for glycan quantification.

### 3.9. Statistical Analysis

Statistical comparisons between two potato cultivars were performed using independent two-sample Student’s *t*-test in OriginPro 2023 software, where the T-statistic and *p*-values were automatically generated. One-way ANOVA combined with Tukey’s post hoc test was adopted for overall comparison among three varieties. Differences with *p* < 0.05 were considered statistically significant, while differences with *p* < 0.01 were regarded as highly significant, and all quantification experiments were conducted with three biological replicates.

## 4. Conclusions

In summary, the present study systematically revealed the cultivar-specific *N*-glycosylation characteristics of patatin from three typical potato cultivars through qualitative and quantitative glycomic analysis. Different potato genotypes exhibited distinct *N*-glycan composition and fucosylation modification patterns in patatin, with significant variations in glycan type distribution and fucosylation abundance across cultivars. The identification of five new *N*-glycans, which have not been documented in previous patatin-related studies, substantially enriches the existing database of plant glycoprotein modification patterns. The unique glycan profile of Favorita, which exclusively contains complex-type *N*-glycans, further demonstrates that the genetic background of potato germplasm profoundly shapes the glycosylation regulatory mechanism of tuber storage proteins. These findings confirm that cultivar difference is a critical factor leading to the structural heterogeneity of patatin *N*-glycosylation, providing a novel perspective for explaining the structural and functional diversity of potato proteins.

From an application perspective, the clarified differential glycosylation features of patatin among different cultivars lay a solid theoretical foundation for the directional selection of high-quality potato germplasms and the targeted development of potato protein processing technologies. Future research can focus on exploring the molecular regulatory mechanism underlying cultivar-specific glycosylation modification, investigating the effects of differential fucosylation patterns on the antioxidant, immunological and functional properties of patatin, and further expanding the research scope to more potato varieties. Such follow-up studies will help fully elucidate the correlation between germplasm characteristics, glycosylation modification and protein functional traits, and promote the high-value utilization of potato protein resources in food and biological industries.

## Figures and Tables

**Table 1:** Summary of the information on patatin *N*-glycans obtained from comprehensive analysis by ESI-MS and online HILIC-MS/MS.

*m*/*z*	Mass	Ion Type	Monosaccharide Composition ^a^	Proposed Structure ^b^	Type	Retention Time (min)	Variety
1373.6 1395.3	1372.3	[M + H]^+^ [M + Na]^+^	Man_3_XylGlcNA_2_ (PMP)_2_	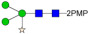	complex	35.87	A, B, C
1357.3	1356.3	[M + H]^+^	Man_2_FucXyl GlcNA_2_(PMP)_2_	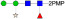	complex	36.41	A, C
1409.6	1386.6	[M + Na]^+^	Man_3_Fuc GlcNAc_2_(PMP)_2_	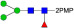	complex	45.61	A
1519.3 1541.3 1557.3	1518.3	[M + H]^+^ [M + Na]^+^ [M + K]^+^	Man_3_FucXyl GlcNAc_2_(PMP)_2_	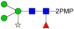	complex	53.50	A, B, C
1558.3	1535.3	[M + Na]^+^	Man_4_Xyl GlcNAc_2_(PMP)_2_	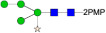	complex	56.81	A
1565.6 1587.2	1564.2	[M + Na]^+^	Man_5_GlcNAc_2_ (PMP)_2_	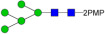	high-mannose	51.74	A
1744.3	1721.3	[M + Na]^+^	Man_3_FucXyl GlcNAc_3_(PMP)_2_	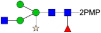	complex	62.05	A
1749.4	1726.4	[M + Na]^+^	Man_6_GlcNAc_2_ (PMP)_2_	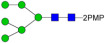	high-mannose	63.08	A, B
1889.5 1911.3	1888.3	[M + H]^+^ [M + Na]^+^	Man_7_GlcNAc_2_ (PMP)_2_	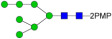	high-mannose	73.35	A, B
1037.3	2050.3	[M + H + Na]^2+^	Man_8_GlcNAc_2_ (PMP)_2_	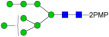	high-mannose	82.58	A, B
1129.3	2212.3	[M + 2Na]^2+^	Man_9_GlcNAc_2_ (PMP)_2_	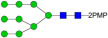	high-mannose	89.16	A, B

^a^ Man, mannose; GlcNAc, *N*-acetylglucosamine; Xyl, xylose; Fuc, fucose. ^b^ Symbol nomenclature: 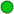, mannose; 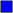, *N*-acetylglucosamine; 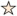, xylose; 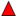, fucose. Gan Nong Shu No. 7 (Wuwei, China) varieties (A), Ke Xin No. 1 (Qiqihar, China) varieties (B) and Favorita (Qingdao, China) varieties (C).

## Data Availability

The original contributions presented in this study are included in the article and Supplementary Materials. Further inquiries can be directed to the corresponding author.

## Supplementary Materials

The following supporting information can be downloaded at: https://www.mdpi.com/article/10.3390/molecules31152591/s1: Figure S1: Representative ESI-MS profiles of patatin N-glycans released by the one-pot chemical method (A) and PNGaseF (B). Symbol nomenclature: , N-acetylglucosamine; , fucose; , xylose; , mannose. Figure S2: MS/MS analysis of N-glycans released from patatin. Symbol nomenclature: , N-acetylglucosamine; , mannose; , fucose; , xylose. Figure S3: EICs of patatin N-glycans. Notes: A is Gan Nong Shu No. 7 (Wuwei, China) varieties, B is Ke Xin No. 1 (Qiqihar, China) varieties and C is Favorita (Qingdao, China) varieties. Symbol nomenclature: , mannose; , N-acetylglucosamine; , xylose; , fucose. Figure S4: Histogram showing the occupancy rates of each N-glycan in different potato protein samples. A is Gan Nong Shu No. 7 (Wuwei, China) varieties, B is Ke Xin No. 1 (Qiqihar, China) varieties and C is Favorita (Qingdao, China) varieties. Man, mannose; GlcNAc, N-acetylglucosamine; Xyl, xylose; Fuc, fucose. Table S1: Summary of the Information of patatin N-Glycans structure assignment Obtained from Comprehensive Analysis.
