# Supplementary material for: Mass Spectrometric Analysis of N-Glycome of Patatin Proteins from Three Potato Cultivars
Source: Molecules. 2026 Jul 24;31(15):2591. doi: 10.3390/molecules31152591 (PMC13468385; doi:10.3390/molecules31152591)
Supplement: Supplementary file 1 [file molecules-31-02591-s001.zip › molecules-4411737-supplementary.pdf]

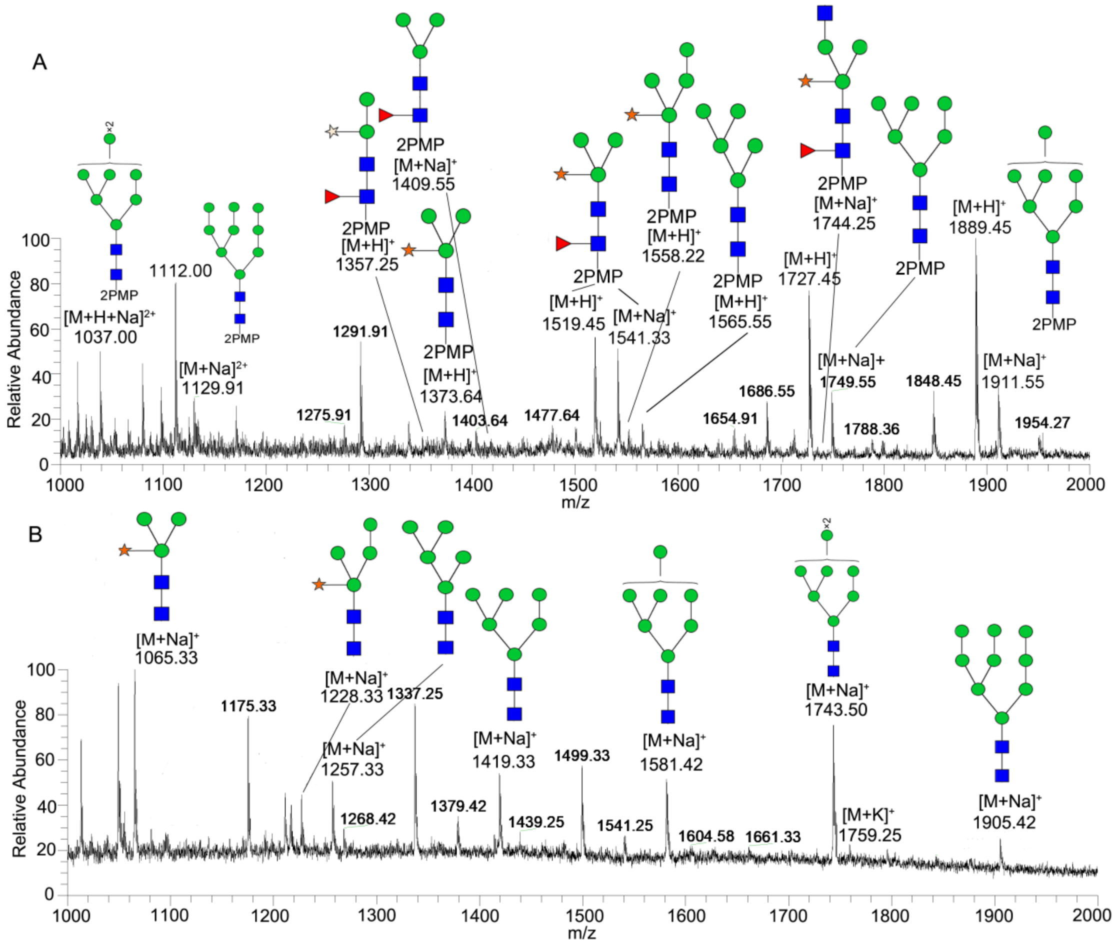

Figure S1 Representative ESI-MS profiles of patatin *N*-glycans released by the one-pot chemical method (A) and PNGaseF (B). Symbol nomenclature: ■, *N*-acetylglucosamine; ▲, fucose; ☆, xylose; ●, mannose.

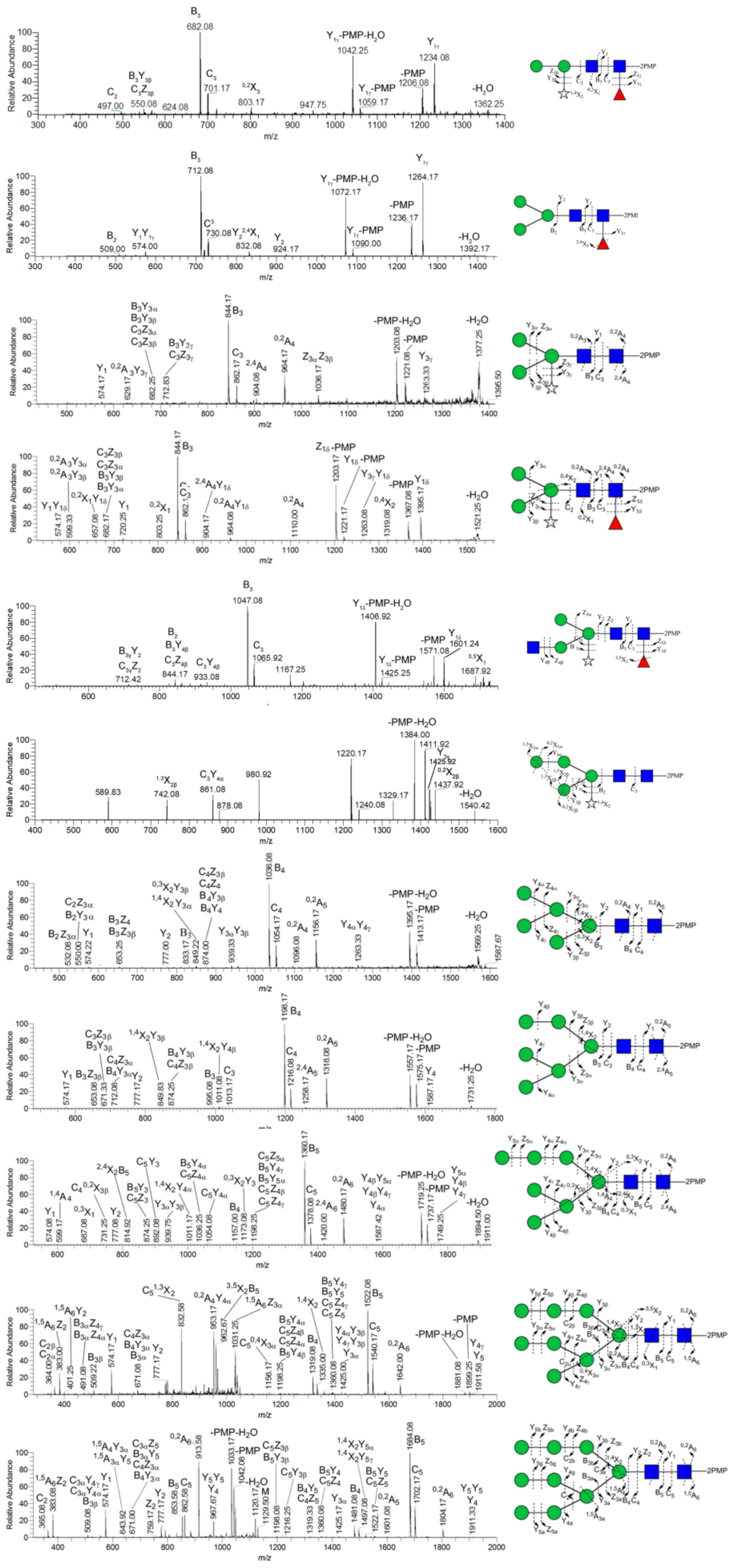

Figure S2 MS/MS analysis of *N*-glycans released from patatin. Symbol nomenclature: ■, *N*-acetylglucosamine; ●, mannose; ▲, fucose; ☆, xylose.

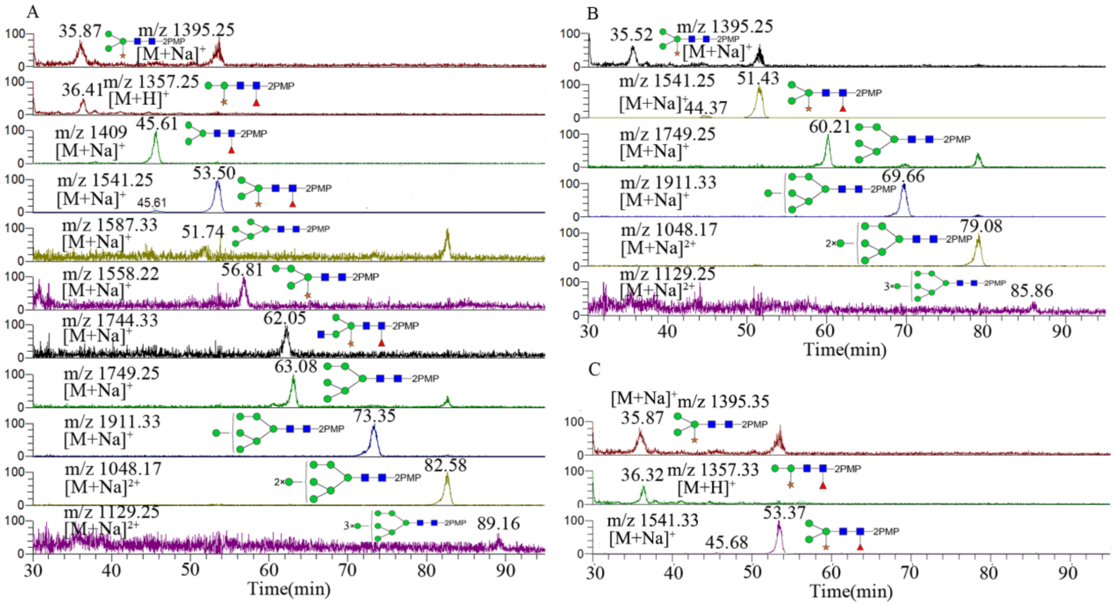

Figure S3 EICs of patatin *N*-glycans. Notes: A is Gan Nong Shu No. 7(Wuwei, China) varieties, B is Ke Xin No.1 (Qiqihar, China) varieties and C is Favorita(Qingdao, China) varieties. Symbol nomenclature: ●, mannose; ■, *N*-acetylglucosamine; ☆, xylose; ▲, fucose.

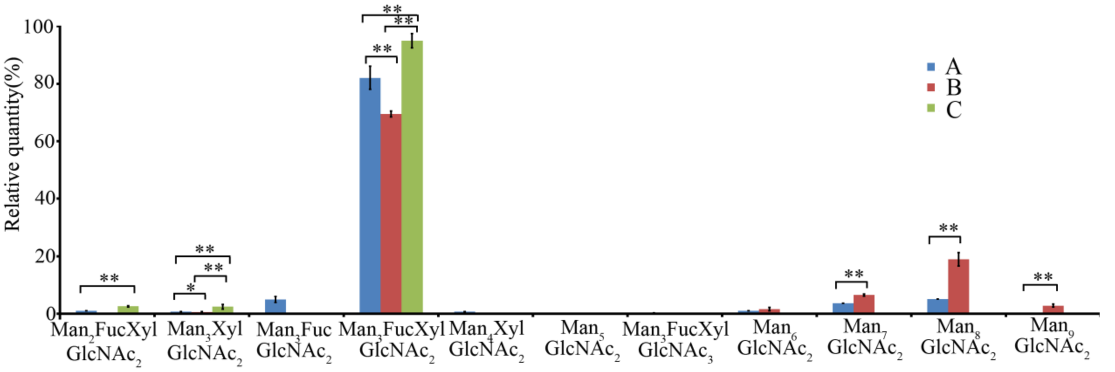

Figure S4 Histogram showing the occupancy rates of each *N*-glycan in different potato protein samples. A is Gan Nong Shu No. 7(Wuwei, China) varieties, B is Ke Xin No.1 (Qiqihar, China) varieties and C is Favorita(Qingdao, China) varieties. Man, mannose; GlcNAc, N-acetylglucosamine; Xyl, xylose; Fuc, fucose.

Table S1. Summary of the Information of patatin *N*-Glycans structure assignment Obtained from Comprehensive Analysis

m/z	Mass	Mass Accuracy (ppm)	Ion Type	Monosaccharide composition[a]	Proposed Structure[b]
1373.64<br>1395.55	1372.64<br>1372.55	-5.68<br>7.31	$[M+H]^+$<br>$[M+Na]^+$	Man$_3$XylG1cNA$_2$ (PMP)$_2$	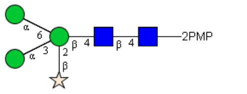
1357.25	1356.25	-5.75	$[M+H]^+$	Man$_2$FucXyl G1cNA$_2$(PMP)$_2$	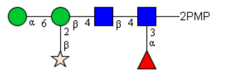

1409.55	1386.55	7.24	[M+Na]$^{+}$	Man$_3$Fuc G1cNAc$_2$(PMP)$_2$	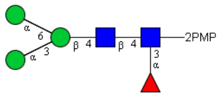
1519.45<br>1541.33<br>1557.30	1518.45<br>1518.33<br>1518.33	-5.13<br>6.62<br>4.05	[M+H]$^{+}$<br>[M+Na]$^{+}$<br>[M+K]$^{+}$	Man$_3$FucXyl G1cNAc$_2$(PMP)$_2$	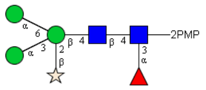
1558.33	1535.33	6.55	[M+Na]$^{+}$	Man$_4$Xyl G1cNAc$_2$(PMP)$_2$	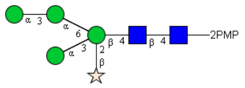
1565.55<br>1587.25	1564.55<br>1564.25	-4.98<br>6.43	[M+Na]$^{+}$	Man$_5$GlcNAc$_2$ (PMP)$_2$	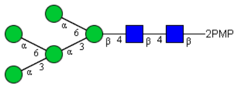
1744.25	1721.25	5.85	[M+Na]$^{+}$	Man$_3$FucXyl G1cNAc$_3$(PMP)$_2$	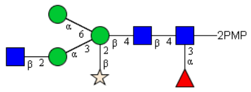
1749.55	1726.55	5.83	[M+Na]$^{+}$	Man$_6$GlcNAc$_2$ (PMP)$_2$	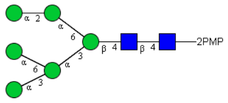
1889.45<br>1911.55	1888.45<br>1888.55	-4.13<br>5.34	[M+H]$^{+}$<br>[M+Na]$^{+}$	Man$_7$GlcNAc$_2$ (PMP)$_2$	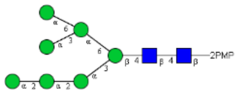
1037.33	2050.66	1.16	[M+H+Na]$^{2+}$	Man$_8$GlcNAc$_2$ (PMP)$_2$	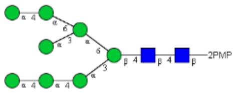
1129.33	2212.66	9.03	[M+2Na]$^{2+}$	Man$_9$GlcNAc$_2$ (PMP)$_2$	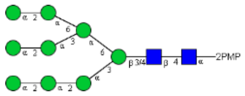

[a]Man, mannose; G1cNAc, *N*-acetylhexosamine; Xyl, xylose; Fuc, fucose. [b]Symbol nomenclature: 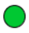, mannose; 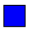, *N*-acetylglucosamine; 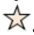, xylose; 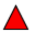, fucose.
